# *Limnospira indica* PCC 8005 Supplementation Prevents Pelvic Irradiation-Induced Dysbiosis but Not Acute Inflammation in Mice

**DOI:** 10.3390/antiox12030572

**Published:** 2023-02-24

**Authors:** Charlotte Segers, Mohamed Mysara, Amelie Coolkens, Shari Wouters, Sarah Baatout, Natalie Leys, Sarah Lebeer, Mieke Verslegers, Felice Mastroleo

**Affiliations:** 1 Interdisciplinary Biosciences Group, Belgian Nuclear Research Centre, SCK CEN, 2400 Mol, Belgium; 2 Department of Bioscience Engineering, University of Antwerp, 2020 Antwerp, Belgium

**Keywords:** ionizing radiation, intestine, mucositis, microbiome, microbiota, dysbiosis, bacterial dietary supplement, radiomitigator

## Abstract

Pelvic irradiation-induced mucositis secondarily leads to dysbiosis, which seriously affects patients’ quality of life after treatment. No safe and effective radioprotector or mitigator has yet been approved for clinical therapy. Here, we investigated the potential protective effects of fresh biomass of *Limnospira indica* PCC 8005 against ionizing irradiation-induced mucositis and dysbiosis in respect to benchmark probiotic *Lacticaseibacillus rhamnosus* GG ATCC 53103. For this, mice were supplemented daily before and after 12 Gy X-irradiation of the pelvis. Upon sacrifice, food supplements’ efficacy was assessed for intestinal barrier protection, immunomodulation and changes in the microbiota composition. While both could not confer barrier protection or significant immunomodulatory effects, 16S microbial profiling revealed that *L. indica* PCC 8005 and *L. rhamnosus* GG could prevent pelvic irradiation-induced dysbiosis. Altogether, our data show that—besides benchmarked *L. rhamnosus* GG—*L. indica* PCC 8005 is an interesting candidate to further explore as a radiomitigator counteracting pelvic irradiation-induced dysbiosis in the presented in vivo irradiation–gut–microbiota platform.

## 1. Introduction

Radiotherapy is commonly used in the clinic to treat a wide variety of cancers. Despite technological improvements, the delivery of ionizing irradiation to a tumor unavoidably affects the surrounding healthy tissues, specifically the rapidly renewing and thus highly radio-sensitive intestine, which is situated within the treatment field for all intra-abdominal, retroperitoneal, as well as pelvic tumors [1,2]. As a consequence, patients often develop acute and chronic intestinal complications, with diarrhea being the most frequently reported symptom [1].

Previously, we reported that, secondarily to the induction of intestinal mucositis, acute pelvic irradiation affected the colonic microbial ecosystem in a mouse model [3]. So-called dysbiosis, defined by an altered composition of microbes having a cascading impact on the immune system and offering an advantage for emergence and outbreak of pathogens [4], may induce a chronic intestinal phenotype [5].

Accordingly, reconstitution or protection of the intestinal ecology following pelvic irradiation may be conferred by food supplements through immunomodulatory and barrier-protective mechanisms as well as intermicrobial interactions [2]. So far, the results of clinical trials have not always been unambiguous, as reviewed earlier [2]. A crucial factor for success might be the appropriate choice of food supplement used.

One of the best-documented model probiotic strains, *Lacticaseibacillus rhamnosus* GG, was reported to exert TLR2/COX2-mediated protection of the intestinal barrier in mice following 12 Gy total body irradiation [6,7]. Additionally, other studies have shown its potential for use as adjunctive therapy in cases of diarrhea [8,9], cancer [10,11] and inflammatory bowel disease [12,13]. These promising results have prompted the initiation of a clinical trial currently exploring its radioprotective potential in patients receiving abdominopelvic irradiation (NCT01790035). Still, its safety, efficacy and mechanism(s) of action remain to be addressed.

Besides traditional probiotic strains, alternative food supplements are being explored for their radioprotective potential. Previously, we introduced anciently used *Limnospira indica* PCC 8005 (also known as *Arthrospira* sp. or its generic product name, Spirulina) [14] because of recent efforts showing beneficial effects of dried biomass and/or isolated bioactive compounds on the intestine’s antioxidant status, immune system and/or bacterial communities [15,16,17,18,19,20]. In 2002, the edible character of *Limnospira* spp. was reviewed by the US Food and Drug Administration and received the GRAS (Generally Recognized as Safe) status. Although no (pre)clinical research investigating the potential of *Limnospira* spp. for reducing radiotherapy-induced intestinal toxicity has yet been conducted, its protective potential has been investigated in other organs. For instance, *Limnospira* sp. (1 g/kg for 15 days) was proposed to ensure nephroprotection in rats after 6.5 Gy total body irradiation by improving the overall lipid profile and reducing oxidative stress and apoptosis [21]. In mice exposed to 2.5 Gy total body irradiation, a crude ethanol precipitate of *Limnospira* sp. (three times 12 mg/kg) was reported to reduce bone marrow damage likely because of transient enhancement of radical-scavenging activity in irradiated cells [22]. The prophylactic and therapeutic potentials of extracted phycocyanin were evaluated in a mouse model exposed to thoracic irradiation in view of pulmonary fibrosis. Both strategies appeared capable of alleviating radiation-induced lung injury when evaluating histology as well as local and systemic inflammatory parameters [23].

To date, the radioprotective potential of unprocessed *L. indica* PCC 8005 on the intestinal ecosystem has not yet been explored in respect to *L. rhamnosus* GG. Therefore, we set out a comparative study in which mice were administered fresh, in-house prepared biomass of either *L. indica* PCC 8005, *L. rhamnosus* GG or saline before and after a single exposure of the pelvis to ionizing radiation.

## 2. Materials and Methods

### 2.1. Mice

All animal experiments were approved by the SCK CEN animal welfare committee and were carried out in accordance with the Ethical Committee Animal Studies of Medanex Clinic (EC MxCl 2018-093), the Belgian laboratory animal legislation and the European Communities Council Directive of 22 September 2010 (2010/63/EU).

Five-week-old, male C57Bl/6JRj mice (Janvier, Bio Services, Uden, The Netherlands) were used, as they were described as one of the most recommended mouse strains for developing radiation countermeasures [24]. Moreover, given the higher incidence of pelvic cancers in males, as reported by the World Health Organization in 2020 (https://gco.iarc.fr/; accessed on 17 December 2022), male mice were chosen. Mice were housed individually in ventilated cages under standard laboratory conditions (12 h light/dark cycle) with ad libitum supply of regular chow and water and were acclimatized for two weeks prior to the start of the experiment.

Confounding factors were minimized across experimental cohorts by randomly assigning mice to a pre-specified number of groups using the *minDiff* package in RStudio (v.3.5.0).

### 2.2. Bacteria and Growth Conditions

*Limnospira indica* PCC 8005 morphotype P1 was obtained from the Pasteur Culture collection of Cyanobacteria (PCC) (Institut Pasteur, France). *L. indica* PCC 8005 cultures were grown axenically in Zarrouk medium (pH ~9.8) at a constant temperature of 30 °C while shaken at 120 rpm [25]. Cells were illuminated at a photon flux density of 45 μmol photons·m^−2^·s^−1^ (Osram, Belgium).

In parallel, *Lacticaseibacillus rhamnosus* GG ATCC 53103, a strain originally isolated from human fecal samples [26,27], was kindly gifted by professor Sarah Lebeer (University of Antwerp). This strain was grown statically in the dark at 37 °C in de Man, Rogosa and Sharpe (MRS) medium (Difco, Belgium) [28].

Fresh bacterial biomass of both species was prepared for supplementation as reported previously [14].

### 2.3. Experimental Setup

**Figure 1:**
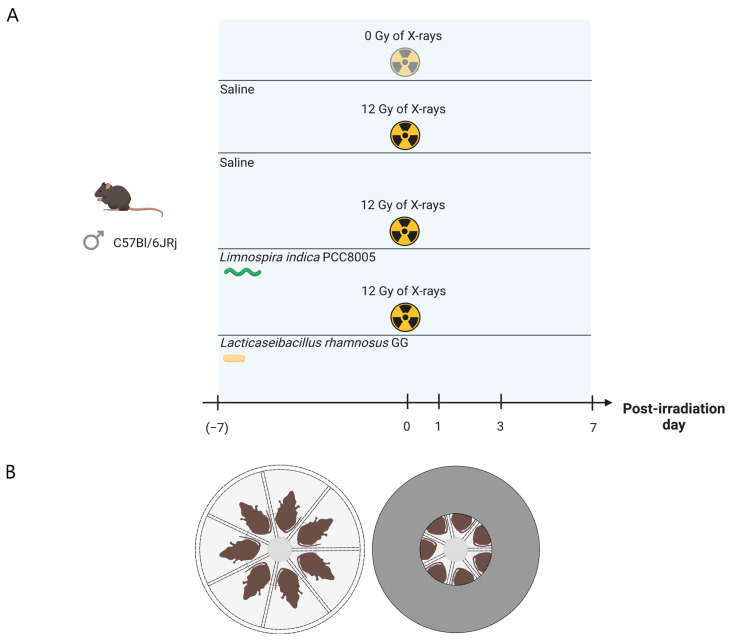
Experimental setup. (**A**) Experimental timeline for supplementation and pelvic irradiation of mice. Supplementation was carried out from seven days prior to pelvic irradiation until the day of sacrifice at either post-irradiation day 1, 3 or 7. Created with BioRender.com. (**B**) Disk-shaped plexiglas box used for local, pelvic irradiation of the mice. Individual animals were placed inside in a prone position (**left**). The entire box, except for the center (9 cm diameter), was shielded with lead (5 mm thick) (**right**). This figure was created with BioRender.com.

Mice were randomly distributed over 4 different groups (*n* = 10 per group); mice receiving (1) sham-irradiation and daily saline (200 μL/mouse); (2) pelvic irradiation and daily saline (200 μL/mouse); (3) pelvic irradiation and daily *L. rhamnosus* GG (~7 × 10^8^ cells/mouse); and (4) pelvic irradiation and daily *L. indica* PCC 8005 (~3 × 10^7^ cells/mouse of 20 g) (Figure 1A). Bacterial suspensions were administered daily by oral gavage in a maximum volume of 10 μL/g body weight starting from seven days prior to pelvic irradiation until the day of sacrifice. The supplementation dose used for *L. rhamnosus* GG was associated with probiotic effects [29,30,31]. The amount of *L. indica* PCC 8005 administered to mice corresponded to a dosing regimen of 800 mg/kg, previously reported to have antioxidative effects in vivo [32,33,34,35,36]. During the entire experimental setup of maximum 14 days, mice were closely monitored for their overall health and body weight. Eventually, all mice were sacrificed at either post-irradiation day (PID) 1, 3 or 7.

### 2.4. Irradiation Protocol

Pelvic irradiation was performed as described earlier [3]. Briefly, eight-week-old mice were anesthetized and placed in a custom-made disk-shaped Plexiglas box, as illustrated in Figure 1B. The entire box, except for the center (9 cm diameter), was shielded with lead (5 mm thick), allowing local pelvic irradiation of the mice (0 Gy or 12 Gy of X-rays). Control (0 Gy) mice were also anesthetized but were not irradiated (i.e., sham-irradiation).

### 2.5. Ileal histology and Histochemistry

We focused on distal ileum, which we previously reported to be more radiosensitive than proximal colon [3]. In detail, tissues were rinsed with ice-cold phosphate-buffered saline (GibcoTM, Thermo Fisher Scientific, Belgium) and fixed in 4% paraformaldehyde (Merck, Belgium). Histology and immunohistochemistry were performed on 5 µm paraffin-embedded tissues cut on a Thermo Scientific HM 340E Electronic Rotary Microtome perpendicular to the long axis of the intestine and mounted onto Superfrost^TM^ microscope slides (Thermo Fisher Scientific, Merelbeke, Belgium).

For Terminal deoxynucleotidyl transferase-mediated dUTP nick end labeling (TUNEL), the In Situ Cell Death Detection Kit (#11684817910 Roche, Merck, Overijse, Belgium) was used according to the manufacturer’s instructions. For visualization, the DAKO Envision+ HRP (DAB, Thermo Fisher Scientific, Belgium) system was used. Slides were imaged using a brightfield Nikon Ti-Eclipse microscope and a 20× objective. The number of positively stained cells were counted in 50 random crypts per mouse (blindly coded) and normalized to the total number of cells in the crypts. In parallel, general morphology of ileal samples was assessed on the same slides by quantifying villus length and crypt depth at 50 random places per mouse (blindly coded).

### 2.6. Ileal Myeloperoxidase Activity Assay

To monitor the degree of acute inflammation, neutrophil myeloperoxidase activity was measured in ileal tissues as previously performed [3]. Data represent units per gram of tissue in which one unit equals the amount of myeloperoxidase necessary to degrade 1 µmol of hydrogen peroxide per minute at 25 °C.

### 2.7. Cytokine Analyses

Total cell lysates from ileal samples were prepared in ice-cold lysis buffer (500 mM NaCl, 50 mM Tris pH 7.5, 2 mM EDTA, 1% Triton X-100, 0.5% sodium deoxycholic acid, 0.1% SDS, 1 mM protease inhibitor). Protein concentrations were determined by bicinchoninic acid assay (Sigma-Aldrich, Overijse, Belgium). Cell extracts were subjected to cytokine analyses using the MSD mouse pro-inflammatory V-plex assay containing antibodies for IL1β, IL10, IFNγ and TNFα. These assays were performed according to manufacturer’s instructions using the MSD Quickplex SQ 120 instrument and MSD Discovery Workbench data analysis software v4.0 (Meso Scale Discovery^TM^; MSD, Rockville, MD, USA).

### 2.8. Western Blot Analysis of Claudin 5

Claudin 5 expression was reported to be positively correlated with barrier integrity [3]. Therefore, total cell lysates from ileal samples were prepared for Western blot analysis as previously performed [3].

### 2.9. Fecal DNA Extraction and 16S rRNA Gene Sequencing

Fecal samples were longitudinally collected every other day, from arrival of mice onwards, to assess the impact of unprocessed *L. indica* PCC 8005 and *L. rhamnosus* GG on the microbiota. Total fecal DNA was extracted and quantified using the DNeasy PowerSoil Pro Kit (Qiagen, Venlo, The Netherlands) and the QuantiFluor dsDNA system (Promega, Leiden, The Netherlands) according to the manufacturer’s instructions. High-throughput amplicon sequencing of the V3-V4 hypervariable region was conducted on BaseClear’s Illumina MiSeq (V3 chemistry) platform according to the manufacturer’s guidelines. Positive and negative controls were included as recommended [37].

### 2.10. Sequencing Data Processing and Analyses

16S rRNA gene sequencing data were processed and analyzed as previously described [3].

### 2.11. Statistical Analyses

Data were processed, analyzed and visualized using RStudio software packages including *ggplot2* and *ggsci*. Outliers, as defined by the Tukey’s fences criteria (i.e., values below Q1 − 1.5 * IQR or above Q3 + 1.5 × IQR), were excluded from further statistical analyses. Statistical significance was determined using linear (mixed) models using the *lme4* package in RStudio unless otherwise mentioned. Differences with *p* < 0.05 were considered statistically significant.

## 3. Results

### 3.1. L. indica PCC 8005 and L. rhamnosus GG Are Unsuccessful in Protecting the Ileal Barrier Damaged by Pelvic Irradiation

**Figure 2:**
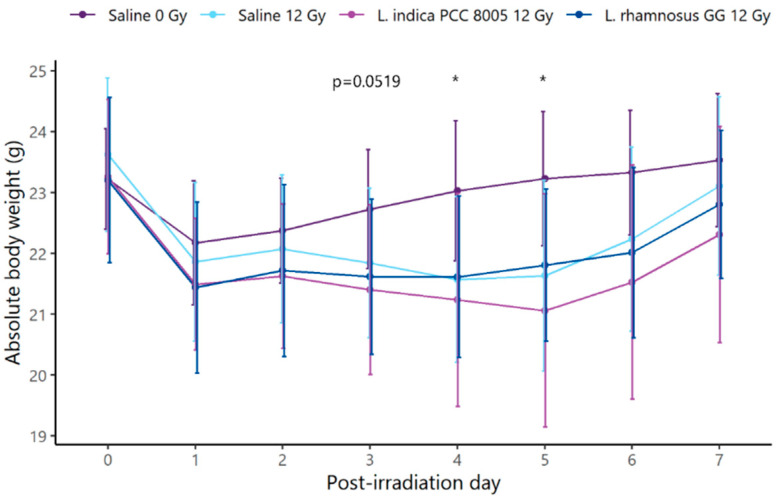
Curve of absolute body weight of all mice subdivided into four treatment groups: saline 0 Gy, saline 12 Gy, *L. indica* PCC 8005 12 Gy and *L. rhamnosus* GG 12 Gy. Data (in grams) are shown as means ± standard deviation, *n* = 10 per group. * *p* < 0.05 for pelvic irradiation-induced differences by linear modeling. Time-dependent differences in respect to the day of (sham-)irradiation (PID0) were not significant as assessed by linear mixed effects modeling.

First, daily monitoring of mice’s body weights showed clear effects of pelvic irradiation (ß = −0.16819; *p* = 0.001544) (Figure 2). Specifically, significant irradiation-induced weight loss was noted at PID4 and PID5. However, supplements could not prevent and/or restore the temporal loss of body weight.

**Figure 3:**
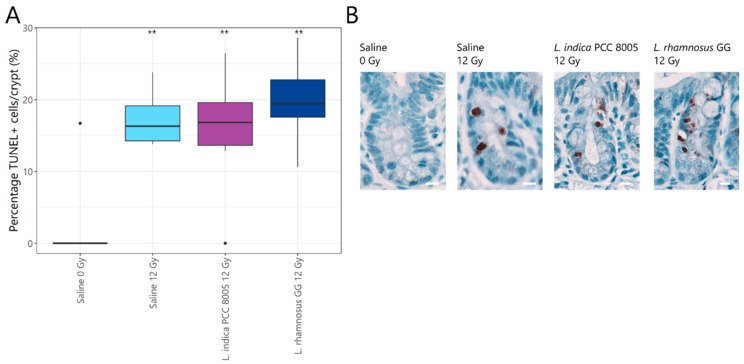
Crypt apoptosis one day following pelvic irradiation. (**A**) Boxplots showing the crypt apoptosis index representing the percentage of TUNEL+ cells per total number of ileal crypt cells one day following (sham-)irradiation. Outliers are depicted by dots, *n* = 10 per group, ** *p* < 0.01 for pelvic irradiation-induced differences in respect to sham-irradiation by Kruskal–Wallis test (Dunn’s post hoc correction). (**B**) Representative images of TUNEL staining obtained at post-irradiation day 1. Brown nuclei are TUNEL+ cells and scale bar represents 10 µm.

**Figure 4:**
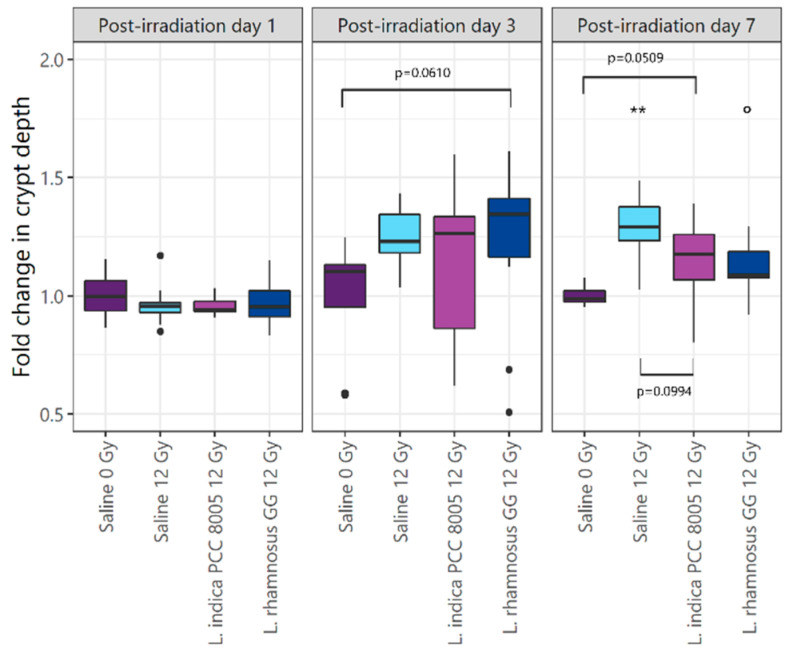
Boxplots showing the fold changes in mucosal crypt depth following pelvic (sham-)irradiation for all different experimental groups. Outliers are depicted by dots, *n* = 10 per group, ** *p* < 0.01 for pelvic irradiation-induced differences in respect to sham-irradiation, and ° *p* < 0.05 for food supplement-dependent differences in respect to saline administration by linear modelling.

Histopathology revealed a rapid, significant increase in the percentage of apoptotic nuclei in all ileal crypts of 12 Gy-exposed mice as compared to sham-irradiated mice (Figure 3A,B). However, both tested supplements appeared unsuccessful in preventing and/or reducing apoptosis induced by pelvic irradiation. A consequent decrease in villus length and crypt depth in response to radiation-induced apoptosis was not observed in our study following local acute pelvic irradiation (Figure 4 and Figure S1). Hence, possible regenerative and/or healing effects of both supplements could not be identified following pelvic irradiation.

**Figure 5:**
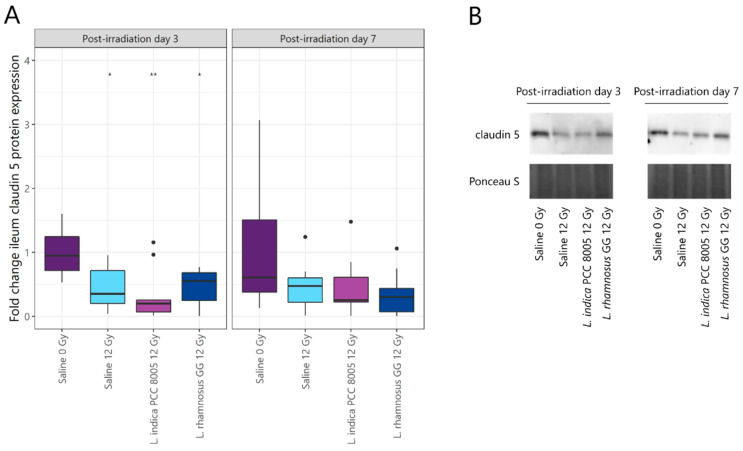
(**A**) Effects of pelvic irradiation on ileum claudin 5 protein expression. Outliers are depicted by dots, *n* = 10 per group. (**B**) Representative Western blot images of claudin 5 (23 kDa) and Ponceau S total protein staining, *n* = 10 per group, * *p* < 0.05 and ** *p* < 0.01 for pelvic irradiation-induced differences in respect to sham-irradiation by Kruskal–Wallis test (Dunn’s post hoc correction).

To assess the consequences of irradiation-induced epithelial apoptosis on mucosal integrity, claudin 5 tight junction expression was investigated by Western blot. Although completely restored at PID7, pelvic irradiation significantly reduced claudin 5 expression at PID3 (Figure 5A,B). Both tested supplements could not intervene in barrier impairment following pelvic irradiation.

### 3.2. L. rhamnosus GG and L. indica PCC 8005 Supplementation Are Not Able to Prevent Acute Ileal Inflammation Induced by Pelvic Irradiation

**Figure 6:**
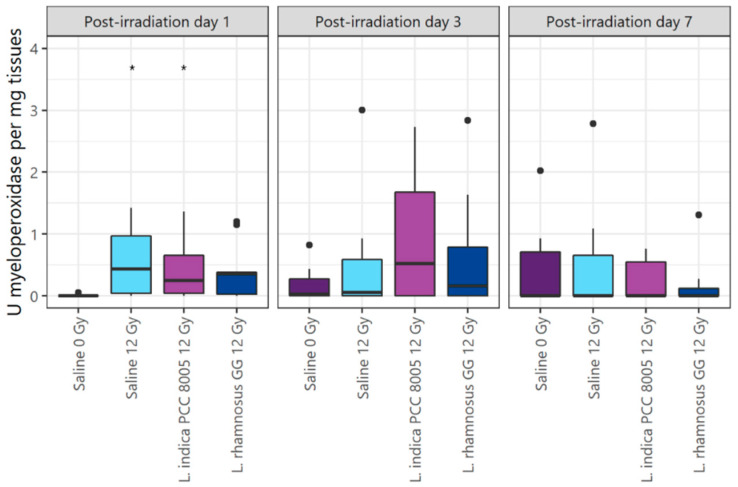
Ileal inflammatory myeloperoxidase activity following (sham-)irradiation. Outliers are depicted by dots, *n* = 10 per group, * *p* < 0.05 for pelvic irradiation-induced differences in respect to sham-irradiation by Kruskal–Wallis test (Dunn’s post hoc correction).

Besides barrier protection, food supplements are being investigated in view of their capacity to modulate the immune response. Therefore, myeloperoxidase activity was measured to monitor mucosal neutrophil inflammation. Molecular analyses showed that pelvic irradiation acutely and temporally provoked an increase in myeloperoxidase activity, which was partly (37%) prevented by *L. rhamnosus* GG supplementation at PID1 (Figure 6).

**Figure 7:**
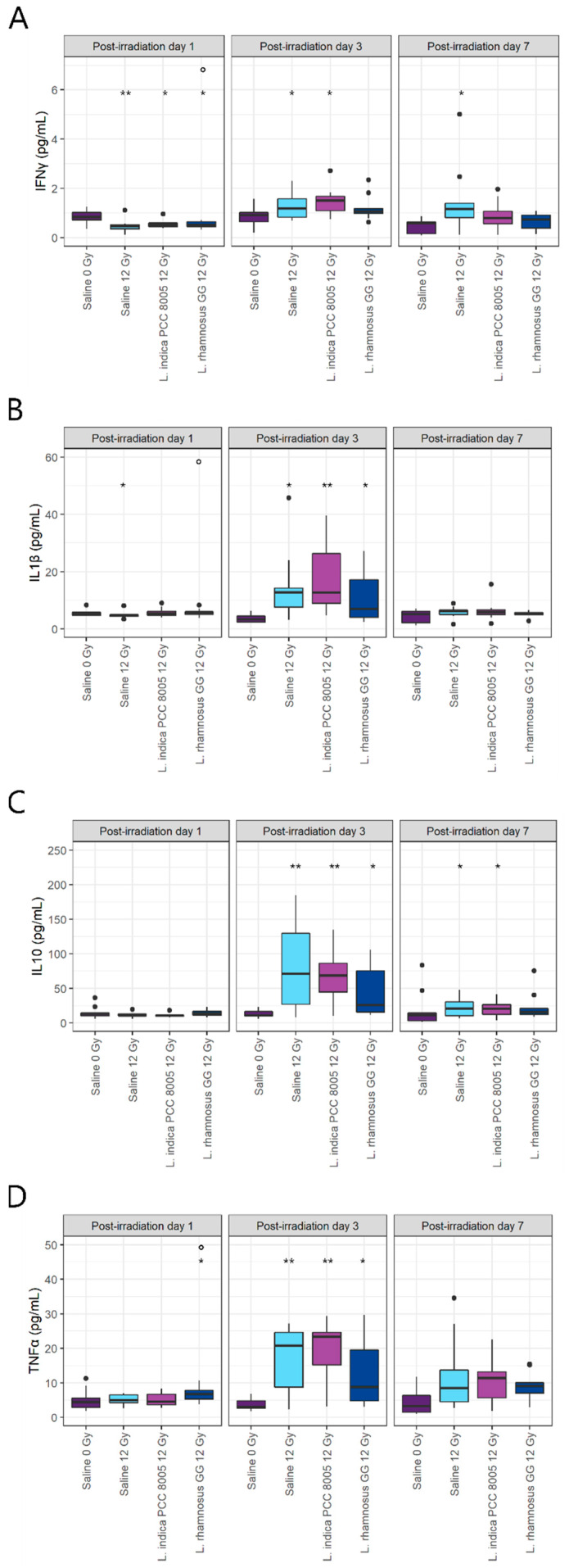
Ileal pro-inflammatory cytokine levels including (**A**) IFNγ, (**B**) IL1β, (**C**) IL10 and (**D**) TNFα following (sham-)irradiation. Outliers are depicted by dots, *n* = 10 per group, * *p* < 0.05 and ** *p* < 0.01 for pelvic irradiation-induced differences in respect to sham-irradiation, and ° *p* < 0.05 for supplementation-induced differences in respect to saline treatment by Kruskal–Wallis test (Dunn’s post hoc correction).

In addition to myeloperoxidase activity, ileal levels of pro-inflammatory cytokines including IFNγ, IL1β, IL10 and TNFα were quantified. Despite a temporal decrease in IFNγ at PID1, pelvic irradiation significantly increased its levels from PID3 onwards (Figure 7A). This latter effect was partly reversed at PID3 and PID7 by *L. rhamnosus* GG supplementation, while IFNγ levels were only restored at PID7 with *L. indica* PCC 8005 supplementation. Both IL1β and IL10 were significantly increased three days following pelvic irradiation (Figure 7B, C). Although none of the tested supplements were capable of lowering these at PID3, IL10 levels partially recovered at PID7 following *L. rhamnosus* GG supplementation (Figure 7C). Finally, pelvic irradiation temporally provoked an increase in TNFα at PID3 (Figure 7D). A modest increase in TNFα was already observed at PID1 for the *L. rhamnosus* GG supplemented group. No restoration of TNFα was observed for either of the tested supplements.

### 3.3. L. indica PCC 8005 and L. rhamnosus GG Prevent Pelvic Irradiation-Induced Dysbiosis

Next, to assess the impact of fresh *L. indica* PCC 8005 and *L. rhamnosus* GG on the irradiated microbiota, feces were collected before and after pelvic exposure for 16S microbial profiling. In this data set, the alpha rarefaction curve and the Good’s estimator of coverage suggested that the 16S rRNA results from each library represented an adequate level of sequencing (Figure S2A,B) [38,39]. Following rarefaction, performed to a depth of ≥6117 reads representing the smallest sample depth, the obtained reads were linked to a total of 694 Operational Taxonomic Units (OTUs) (167 ± 16 OTUs on average per sample).

**Figure 8:**
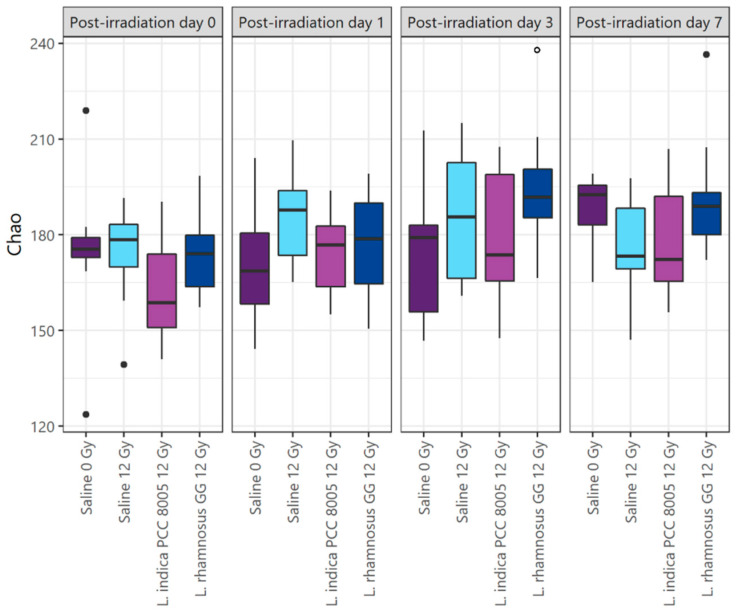
Changes in alpha diversity index Chao, considering solely richness, following (sham-)irradiation. Data are presented in boxplots and outliers are depicted by dots, *n* = 10 per group. Time-independent differences were assessed by Mann–Whitney’s U test (Bonferroni’s post hoc correction). Time-series, pairwise comparisons in respect to post-irradiation day 0 using ° *p* <0.05 were performed by Wilcoxon’s signed rank test (Bonferroni’s *post-hoc* correction).

Changes in the microbial communities introduced by pelvic irradiation, and the different food supplements tested were evaluated using microbial alpha and beta diversity indices. At indicated sampling points, no irradiation- nor supplementation-induced effects on alpha diversity metrics were observed (Chao, Shannon, Shannon even indices; Figure 8, Figures S3 and S4). However, paired comparisons of alpha diversity metrics in respect to corresponding baseline (PID0) revealed a temporally increased richness at PID3 for mice supplemented with *L. rhamnosus* GG (Chao index; Figure 8).

Microbiota community structures were further compared by a distance matrix based on unweighted (considering microbial membership) UniFrac beta diversity index with 1000 permutations. At baseline (at PID0), a significant shift of unweighted UniFrac beta diversity was noted when comparing both supplemented microbial communities to each other (Table S1 and Figure S5A). While differences were no longer detected at PID1 and PID3, shifts in microbial communities re-appeared at PID7 (Table S1 and Figure S5B–D).

**Figure 9:**
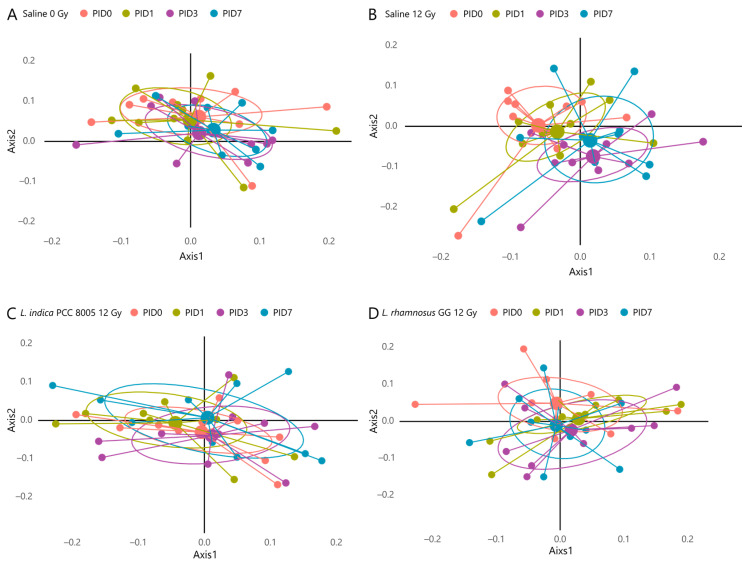
Unweighted UniFrac NMDS plots of (**A**) Saline 0 Gy-, (**B**) Saline 12 Gy-, (**C**) *L. indica* PCC 8005 12 Gy- and (**D**) *L. rhamnosus* GG 12 Gy-administered mice showing the beta diversity between samples, *n* ≥ 9 per group. PID = post-irradiation day.

To exclude the compositional differences present at PID0 introduced by the different supplementation regimens, analyses were performed in a paired manner, capturing the individual changes for each mouse over time. For saline-administered mice exposed to pelvic irradiation, analyses revealed a significant shift in unweighted UniFrac beta diversity at PID3 and PID7 in respect to its microbial community before pelvic irradiation (PID0) (Table 1 and Figure 9). When analyzing the other cohorts, including Saline 0 Gy, *L. indica* PCC 8005 12 Gy and *L. rhamnosus* GG 12 Gy, no significant shifts could be detected over time (Table 1 and Figure 9).

**Figure 10:**
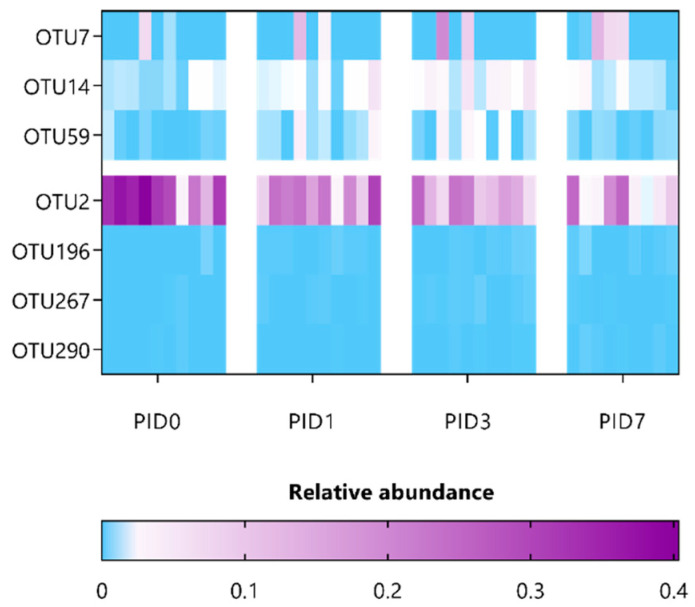
Heatmap representing the relative abundance of significant gut microbial dysbiosis markers detected in *L. indica* PCC 8005-given mice following sham-irradiation as listed in Table 2, *n* = 10 per time point. OTU = operational taxonomic unit; PID = post-irradiation day.

**Figure 11:**
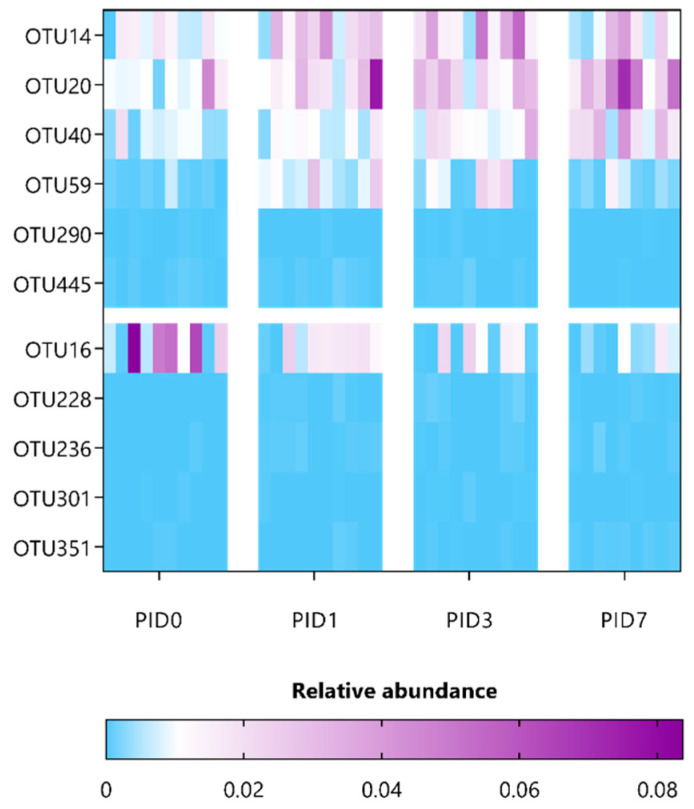
Heatmap representing the relative abundance of significant gut microbial dysbiosis markers detected *L. rhamnosus* GG-given mice following sham-irradiation as listed in Table 3, *n* = 10 per time point. OTU = operational taxonomic unit; PID = post-irradiation day.

Next, to correlate specific OTUs with pelvic irradiation and/or supplementation, the composition of fecal microbiota was investigated in a paired manner using ANCOM and then further refined using oligotyping. A minor list of only three OTUs was identified for saline-administered, sham-irradiated mice (Table S2 and Figure S6), thus indicating a rather stable microbial community over time. In contrast, 13 OTUs were listed to be differentially affected in saline-administered, irradiated mice and belong to the *Porphyromonadaceae* and *Lachnospiraceae* families, including OTU135 (identified as *Alistipes putredinis*) (Table S3 and Figure S7). Furthermore, supplementation with either *L. indica* PCC 8005 or *L. rhamnosus* GG differentially impacted 7 and 11 OTUs, respectively (Table 2 and Table 3 and Figure 10 and Figure 11). Of interest, three significant OTUs were shared between the latter supplementation groups, including OTU14 (*Bacteroidales* spp.) and OTU59 (*Ruminococcaeae* spp.), as well as OTU290 (*Ruminococcaeae* spp.), which were introduced or reduced following pelvic irradiation, respectively. In particular, mice supplemented with *L. indica* PCC 8005 predominantly lost OTUs belonging to the *Porphyromonadaceae* and *Ruminococcaceae* family, while OTU7 (identified as *Akkermansia muciniphila*) was significantly increased in some mice (Table 2 and Figure 10). For mice supplemented with *L. rhamnosus* GG, members belonging to the *Porphyromonadaceae* family were elevated (Table 3 and Figure 11).

## 4. Discussion

**Figure 12:**
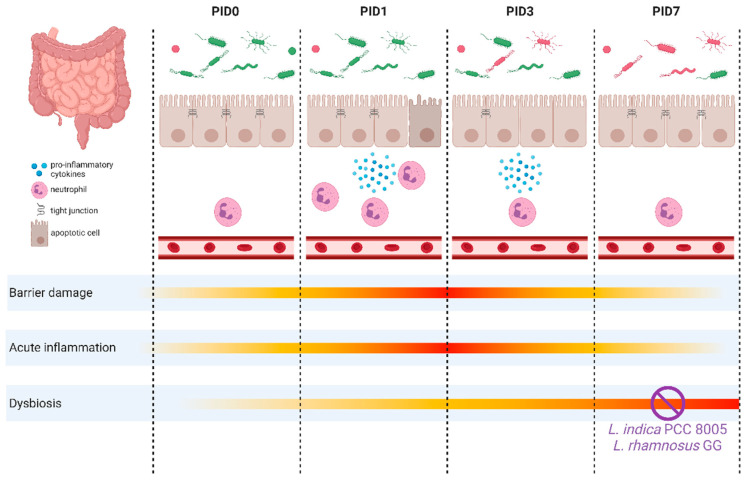
Graphical summary of the main results illustrating the impact of pelvic irradiation on the intestinal ecology based on the different parameters monitored in this study. Pelvic irradiation revealed morphological and inflammatory implications. Concurrent 16S microbial profiling showed a delayed impact of pelvic irradiation on the microbiota composition, which was prevented by both *L. indica* PCC 8005 and *L. rhamnosus* GG. This figure was created with BioRender.com and adapted from [3]. PID = post-irradiation day.

Pelvic radiotherapy has commonly been associated with intestinal complications. Previously, we described the pathobiology of ileal mucositis in mice following pelvic irradiation [3]. Briefly, a primary damage response was rapidly initiated with apoptosis and inflammation, as shown by histology and myeloperoxidase activity, respectively. Amplification of these destructing signals impaired the barrier integrity, characterized by loss of tight junctions, which provides the opportunity for luminal bacteria to translocate into mesenteric lymph nodes. Hereafter, secondarily to these structural and functional changes, a dysbiotic microbial community developed, as summarized in Figure 12.

Among other—technical and biological—strategies, nutritional interventions including vitamins, prebiotics and probiotics have been explored to reconstitute and/or protect the intestinal ecology following pelvic irradiation, as reviewed earlier [2]. To exert beneficial effects, they are thought to intervene in barrier protection and immunomodulation as well as to interact with the microbial community. Unfortunately, (pre-)clinical evidence for the prevention and/or reduction of irradiation-induced intestinal toxicities has not always been unambiguous. The utmost challenge to be overcome concerns the inter-individual response attributed to the variability in patients’ microbial profiles. Herein, the selection of an appropriate personalized therapy might provide a solution. Accordingly, apart from traditional probiotic strains including *L. rhamnosus* GG, alternative food supplements should be explored. Here, the radioprotective efficacy of fresh and unprocessed *L. indica* PCC 8005 biomass was studied in respect to benchmarked *L. rhamnosus* GG.

At first, both supplements were studied for their barrier-protective effects following pelvic irradiation, which was shown to drastically impair the barrier by increasing oxidative stress as well as epithelial cell death and permeability [3,40]. The research group of Stenson et al. showed that administration of *L. rhamnosus* GG steered the migration of mesenchymal stem cells to small intestinal crypts to support epithelial cell proliferation and re-enforce the small intestinal barrier following irradiation [6,7]. In contrast to these reports, *L. rhamnosus GG* appeared unsuccessful in preventing and/or restoring epithelial cells and/or tight junction proteins, likely due to the dramatic effects introduced by total body irradiation [6,7] as compared to acute local pelvic irradiation in our study. Additionally, *Limnospira* sp.-mediated activation of the antioxidant defense system, including glutathione-s-transferase, superoxide dismutase and catalase, is well-described for daily pure phycocyanin (50 mg/kg) supplementation [41,42]. However, significant interference in oxidative stress and subsequent cell death, or loss of epithelial tight junctions induced by pelvic irradiation, could not be observed in our study. This might be explained by the early degradation of *L. indica* PCC 8005 biomass within the acid environment of the intestinal tract or the lower pigment content of the used biomass, both resulting in a lesser amount of antioxidant phycocyanin reaching the ileum.

Besides barrier protection, food supplements are commonly evaluated based on their immunomodulatory capacities. Our data indicate that *L. rhamnosus* GG appears to only be capable of partly reducing neutrophilic myeloperoxidase activity and IFNγ secretion, while elsewhere *L. rhamnosus* GG was shown to prevent epithelial barrier dysfunction induced by IFNγ in human intestinal enteroids and colonoids [43]. In contrast, *L. indica* PCC 8005 could not be linked with such acute anti-inflammatory effects in our experimental setup. Inconsistent outcomes have been reported for myeloperoxidase amelioration following *Limnospira* spp. supplementation in inflammatory mouse models [16,44].

Finally, with regard to the specific impact of food supplements *L. indica* PCC 8005 and *L. rhamnosus* GG on the host’s microbiota, both seem to introduce supplement-specific changes in the microbial community. Here, we could report a temporally increased richness due to *L. rhamnosus* GG supplementation. Paired beta analysis—ruling out the inter-group variability at start—confirmed a delayed, yet significant, shift for saline-administered, irradiated mice, as reported earlier [3], which was impeded by both *L. indica* PCC 8005 and *L. rhamnosus* GG supplements. This indicates that a stable, yet different, microbiota is introduced by both supplementation regimens, which could not be disturbed by acute pelvic irradiation.

Then, unraveling relevant OTUs showed that *Alistipes putredinis* was increased by *L. indica* PCC 8005 supplementation in relative abundance following pelvic irradiation. Interestingly, *Alistipes* spp. were recently reviewed for their high relevance in dysbiosis and intestinal diseases, which can be either beneficial or harmful [45]. After daily supplementation, both *L. indica* PCC 8005 and *L. rhamnosus* GG do not seem to establish themselves in significant numbers in the murine gut. However, *L. rhamnosus* GG has been proven to survive intestinal passage [27,46,47], whereas no such evidence could be found for the highly digestible biomass of *Limnospira* spp. Nevertheless, changes in microbial members were noted for both, highlighting the non-necessity of bacterial supplements to colonize the gut in order to exert beneficial effects, as suggested by other researchers [46,48]. One possible explanation for these effects on the intestinal microbial community might be metabolic cross-feeding [49]. In *L. indica* PCC 8005-supplemented mice, an increased relative abundance of *Akkermansia muciniphila* was detected in some (30%) mice, in accordance with previous research [48,50]. Although the *Akkermansia* genus is widely studied for its promising next-generation probiotic potential, their broad effects on the host beyond their therapeutic niche require careful holistic investigations. For instance, better strain-level identification of human bacteria will help unravel some of this complexity [51]. Interestingly, *Limnospira* spp. are hypothesized to deliver a range of prebiotics including carbohydrates, polyphenols and polyunsaturated fatty acids, which may stimulate growth of beneficial bacteria exerting secondary health benefits such as antioxidative defense [18]. These data might suggest that longer and even combined supplementation of *L. indica* PCC 8005 and *L. rhamnosus* GG might be required to complete this path. To assess the consequences of both supplementation regimens, future research might focus on fecal metabolites to validate the mechanisms of cross-feeding between these taxa.

Despite their promising radiomitigating potential, supplementation of *L. indica* PCC 8005 or *L. rhamnosus* GG fresh biomass could not be associated with barrier protective or significant immunomodulatory effects. Stabilization of the microbial community members and thus prevention of pelvic irradiation-induced dysbiosis was observed for both of the supplementation regimens. The present study concludes that—besides benchmarked *L. rhamnosus* GG—*L. indica* PCC 8005 is an interesting candidate to further investigate how to counteract pelvic irradiation-induced dysbiosis.

## Figures and Tables

**Table 1:** *p* values (** *p* <0.01) of paired unweighted UniFrac beta diversity analyses by AMOVA. PID = post-irradiation day.

	Unweighted UniFrac Beta Diversity
Saline 0 Gy	Overall *p* value: 0.453
Saline 12 Gy	Overall *p* value: 0.006 ** Post-hoc p values: PID0 vs. PID1 *p* = 0.658 PID0 vs. PID3 *p* = 0.004 ** PID0 vs. PID7 *p* = 0.001 ** PID1 vs. PID3 *p* = 0.197 PID1 vs. PID7 *p* = 0.006 ** PID3 vs. PID7 *p* = 0.008 **
*L. indica* PCC 8005 12 Gy	Overall *p* value: 0.124
*L. rhamnosus* GG 12 Gy	Overall *p* value: 0.422

**Table 2:** Differential operational taxonomic units (OTUs) detected in *L. indica* PCC 8005-given mice following pelvic irradiation. PID = post-irradiation day.

Taxonomic Classification (Following Ribosomal Database Project)	ANCOM Biomarkers’ Effect Size and *W*-Statistic	Highest NCBI Blast Hit (% Identity)
*Akkermansia*_OTU7	1.03; *W* = 0.9 (PID7)	*Akkermansia muciniphila* (~100% identity)
*Bacteroidales*_OTU14	1.58; *W* = 0.9 (PID3)	
*Ruminococcus*_OTU59	1.57; *W*= 0.7 (PID3)	
*Porphyromonadaceae*_OTU2	−1.21; *W* = 0.9 (PID7)	
*Ruminococcaceae*_OTU196	−1.21; *W* = 0.9 (PID1)	
*Ruminococcaceae*_OTU267	−1.10; *W* = 0.9 (PID3)	
*Ruminococcaceae*_OTU290	−1.16; *W* = 0.9 (PID7)	

**Table 3:** Differential operational taxonomic units (OTUs) detected in *L. rhamnosus* GG-given mice following pelvic irradiation. PID = post-irradiation day.

Taxonomic Classification (Following Ribosomal Database Project)	ANCOM Biomarkers’ Effect Size and *W*-Statistic	Highest NCBI Blast Hit (% Identity)
*Bacteroidales*_OTU14	1.14; *W* = 0.8 (PID3)	
*Porphyromonadaceae*_OTU20	1.27; *W* = 0.9 (PID7)	
*Porphyromonadaceae*_OTU40	1.41; *W* = 0.7 (PID7)	
*Ruminococcus*_OTU59	2.15; *W* = 0.8 (PID1); 1.43; *W* = 0.8 (PID3)	
*Ruminococcaceae*_OTU290	1.01; *W* = 0.9 (PID1)	
*Lachnospiraceae*_OTU445	1.09; *W*= 0.9 (PID7)	*Muricomes intestine* (>97% identity)
*Lachnospiraceae*_OTU16	−1.31; *W* = 0.9 (PID7)	
*Firmicutes*_OTU228	−1.06; *W* = 0.9 (PID1); −1.17; *W* = 0.9 (PID7)	
*Firmicutes*_OTU236	−1.20; *W* = 0.9 (PID1)	
*Lachnospiraceae*_OTU301	−1.07; *W* = 0.9 (PID3)	
*Lachnospiraceae*_OTU351	−1.17; *W* = 0.9 (PID7)	

## Data Availability

The data presented in this study are available on request from the corresponding author.

## Supplementary Materials

The following supporting information can be downloaded at: https://www.mdpi.com/article/10.3390/antiox12030572/s1, Figure S1: Boxplots showing the fold changes in mucosal parameter villus length following pelvic (sham-)irradiation for all different experimental groups; Figure S2: An adequate depth of sequencing was reached to identify most diversity in the samples; Figure S3: Changes in alpha diversity index Shannon; Figure S4: Changes in alpha diversity index Shannon even, considering solely evenness, following (sham-)irradiation; Figure S5: Unweighted UniFrac NMDS plots; Figure S6: Heatmap representing the relative abundance of significant gut microbial dysbiosis markers detected in saline-given mice following sham-irradiation; Figure S7: Heatmap representing the relative abundance of significant gut microbial dysbiosis markers detected in saline-given mice following pelvic irradiation; Table S1: *p* values of conditional unweighted UniFrac beta diversity analyses by AMOVA; Table S2: Differential OTUs detected in saline-given mice following sham-irradiation; Table S3: Differential OTUs detected in saline-given mice following pelvic irradiation.
