# Supplementary material for: Limnospira indica PCC 8005 Supplementation Prevents Pelvic Irradiation-Induced Dysbiosis but Not Acute Inflammation in Mice
Source: Antioxidants (Basel). 2023 Feb 24;12(3):572. doi: 10.3390/antiox12030572 (PMC10045453; doi:10.3390/antiox12030572)
Supplement: Supplementary file 1 [file antioxidants-12-00572-s001.zip › antioxidants-2139992-supplementary.pdf]

antioxidants

MDPI

Supplementary Materials

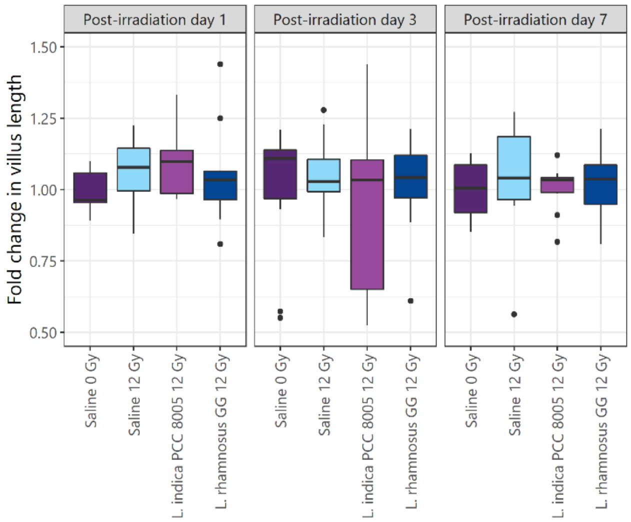

Figure S1. Boxplots showing the fold changes in mucosal parameter villus length following pelvic (sham-)irradiation for all different experimental groups, n = 10 per group. Statistical differences were assessed by linear modelling.

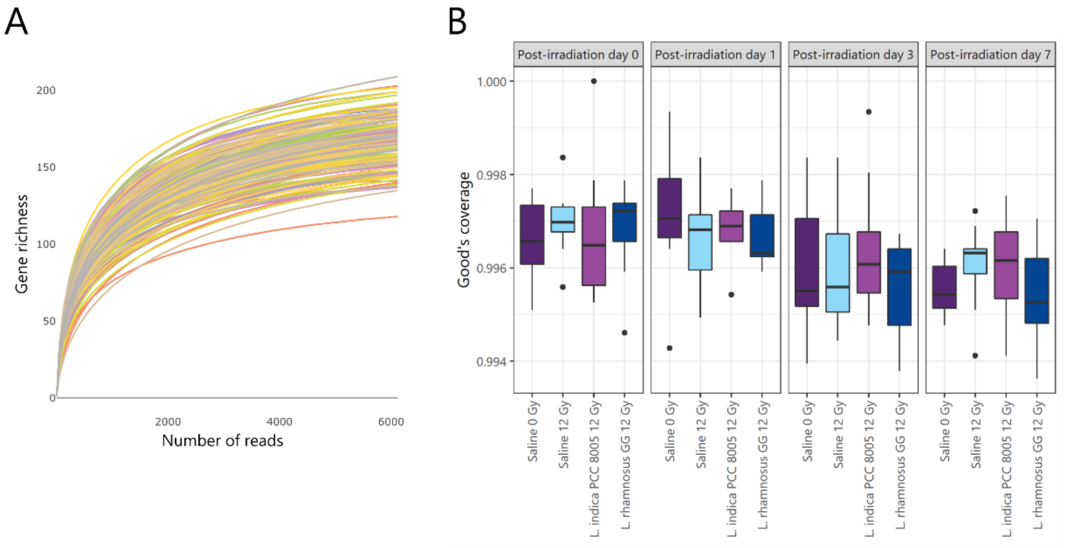

Figure S2. An adequate depth of sequencing was reached to identify most diversity in the samples. (**A**) Rarefaction curve displaying gene richness as a function of the number of reads per sample. (**B**) Good's estimator of coverage as a measure of sample completeness.

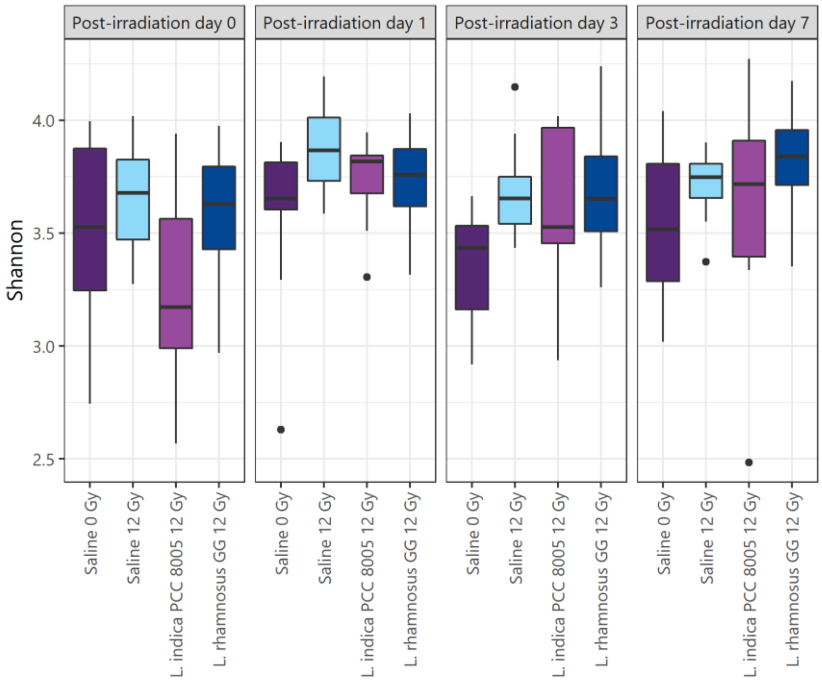

Figure S3. Changes in alpha diversity index Shannon, considering both richness and evenness, following (sham-)irradiation. Data are presented in boxplots, *n* = 10 per group. Time independent and –dependent differences were assessed by Mann Whitney U test (Bonferroni's post-hoc correction) and Wilcoxon signed rank test (Bonferroni's post-hoc correction), respectively.

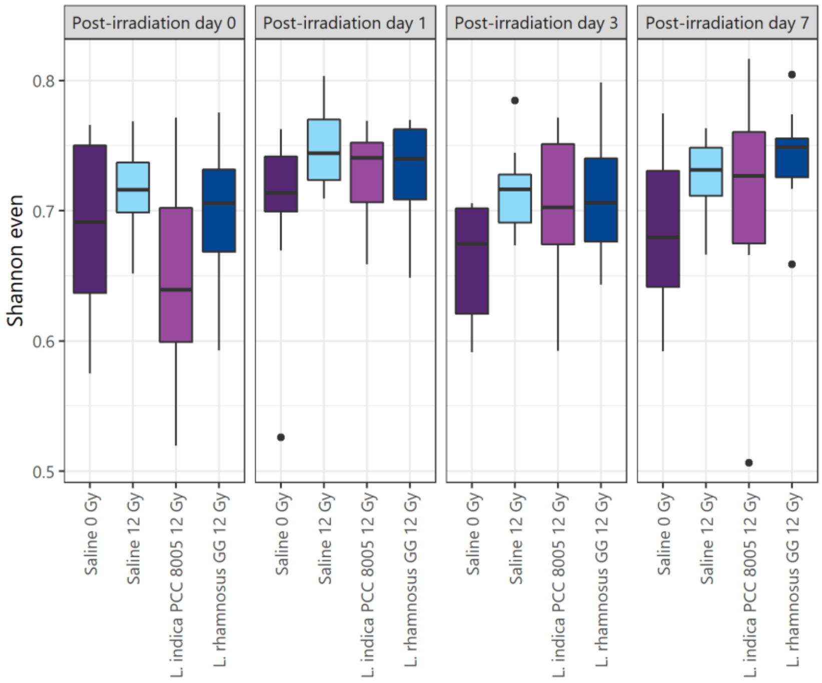

Figure S4. Changes in alpha diversity index Shannon even, considering solely evenness, following (sham-) irradiation. Data are presented in boxplots, *n* = 10 per group. Time independent and –dependent differences were assessed by Mann Whitney U test (Bonferroni's post-hoc correction) and Wilcoxon signed rank test (Bonferroni post-hoc correction), respectively.

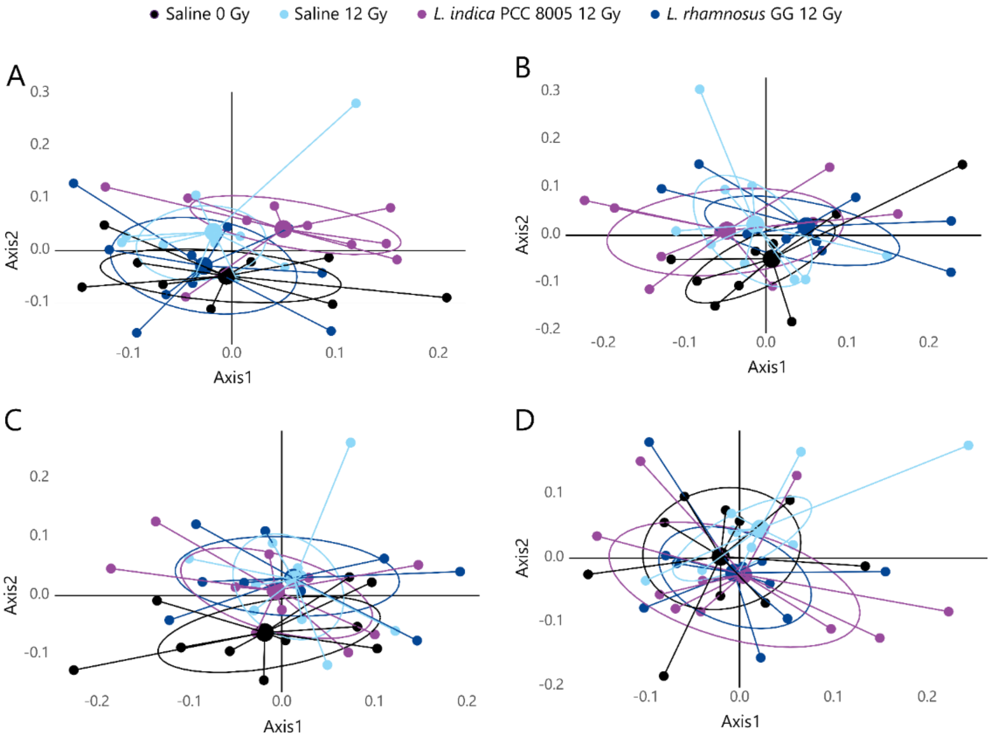

Figure S5. Unweighted UniFrac NMDS plots displaying inter-sample diversity among the different treatment groups at (**A**) post-irradiation day 0, (**B**) post-irradiation day 1, (**C**) post-irradiation day 3 and (**D**) post-irradiation day 7, $n \geq 9$ per group.

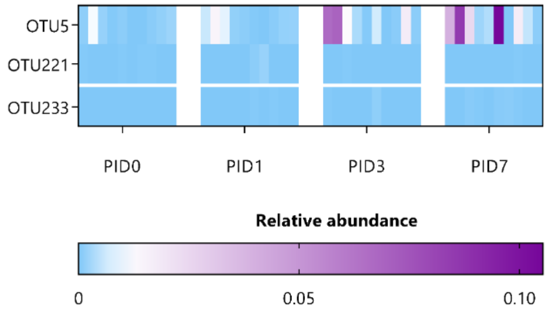

Figure S6. Heatmap representing the relative abundance of significant gut microbial dysbiosis markers detected in saline given mice following sham-irradiation as listed in Supplementary table 2, n = 10 per time point. PID = post-irradiation day.

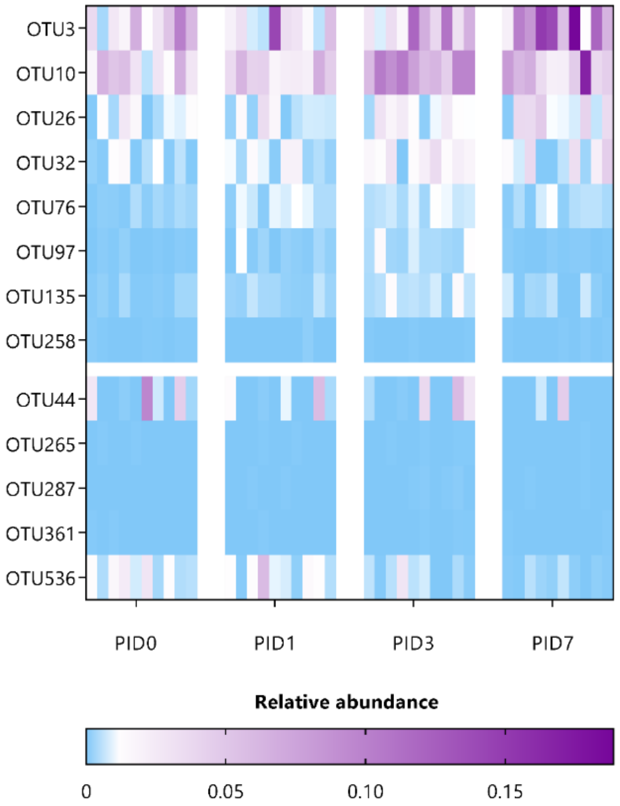

Figure S7. Heatmap representing the relative abundance of significant gut microbial dysbiosis markers detected in saline given mice following pelvic irradiation as listed in Supplementary table 3, n = 10 per time point. PID = post-irradiation day.

Table S1. p values (°p < 0.1, * p <0.05, ** p <0.01) of conditional unweighted UniFrac beta diversity analyses by AMOVA.

	**Unweighted Unifrac Beta Diversity**
**PID0**	Overall p value: 0.015 *<br>*Post-hoc p* values:<br>• Saline 12 Gy vs Saline 0 Gy p = 0.244<br>• *L. indica* PCC 8005 12 Gy vs Saline 0 Gy p = 0.054 °<br>• *L. rhamnosus* GG 12 Gy vs Saline 0 Gy p = 0.16<br>• *L. indica* PCC 8005 12 Gy vs Saline 12 Gy p = 0.179<br>• *L. rhamnosus* GG 12 Gy vs Saline 12 Gy p = 0.125<br>• *L. rhamnosus* GG 12 Gy vs *L. indica* PCC 8005 12 Gy p = 0.007 *
**PID1**	Overall p value: 0.235
**PID3**	Overall p value: 0.408
**PID7**	Overall p value: 0.008 *<br>*Post-hoc p* values:<br>• Saline 12 Gy vs Saline 0 Gy p = 0.077 °<br>• *L. indica* PCC 8005 12 Gy vs Saline 0 Gy p = 0.015 *<br>• *L. rhamnosus* GG 12 Gy vs Saline 0 Gy p = 0.034 *<br>• *L. indica* PCC 8005 12 Gy vs Saline 12 Gy p = 0.279<br>• *L. rhamnosus* GG 12 Gy vs Saline 12 Gy p = 0.132<br>• *L. rhamnosus* GG 12 Gy vs *L. indica* PCC 8005 12 Gy p = 0.79

Table S2. Differential OTUs detected in saline given mice following sham-irradiation.

Taxonomic Classification (Following Ribosomal Database Project)	ANCOM Biomarkers' Effect Size and *W*-Statistic	Highest NCBI Blast Hit (% Identity)
*Erysipelotrichaceae*_OTU5	1.47; *W* = 0.9 (PID3); 2.15; *W* = 0.9 (PID7)	*Faecalibaculum rodentium* (> 97% identity)
*Coriobacteriaceae*_OTU221	1.06; *W* = 0.9 (PID1); 1.06; *W* = 0.9 (PID3); 1.06; *W* = 0.9 (PID7)	
*Firmicutes*_OTU233	−1.27; *W* = 0.9 (PID7)	

Table S3. Differential OTUs detected in saline given mice following pelvic irradiation.

Taxonomic Classification (Following Ribosomal Database Project)	ANCOM Biomarkers' Effect Size and *W*-Statistic	Highest NCBI Blast Hit (% Identity)
*Lactobacillus*_OTU3	1.10; *W*= 0.9 (PID7)	*Lactobacillus taiwanensis* (> 99% identity)
*Prevotella*_OTU10	1.16; *W* = 0.9 (PID3)	
*Bacteroides*_OTU26	1.10; *W* = 0.9 (PID7)	*Bacteroides stercorirosoris* (> 98% identity)
*Porphyromonadaceae*_OTU32	1.04; *W* = 0.9 (PID1); 2.03; *W* = 0.9 (PID3); 1.51; *W* = 0.9 (PID7)	
*Porphyromonadaceae*_OTU76	1.08; *W* = 0.7 (PID3); 1.03; *W* = 0.7 (PID7)	
*Rhodospirillaceae*_OTU97	2.01; *W* = 0.8 (PID3)	
*Alistipes*_OTU135	1.90; *W* = 0.8 (PID3)	*Alistipes putredinis* (~97% identity)
*Clostridiales*_OTU258	1.09; *W* = 0.9 (PID1)	
*Lachnospiraceae*_OTU44	−1.14; *W* = 0.9 (PID7)	
*Ruminococcaceae*_OTU265	−1.16; *W* = 0.9 (PID3)	
*Bacteria*_OTU287	−1.05;*W* = 0.9 (PID3)	
*Lachnospiraceae*_OTU361	−1.12; *W* = 0.9 (PID3)	
*Lachnospiraceae*_OTU536	−1.84; *W* = 0.8 (PID7)	
